# Animal Resources in the Economy of Medieval Moldova: Archaeozoological Case Study of the Urban Settlement from Târgu Neamț (NE Romania)

**DOI:** 10.3390/ani13142334

**Published:** 2023-07-17

**Authors:** Margareta Simina Stanc, Luminița Bejenaru, Mariana Popovici, Vasile Diaconu, Mihaela Danu

**Affiliations:** 1 Faculty of Biology, Alexandru Ioan Cuza University of Iasi, 700505 Iasi, Romania; simina.stanc@uaic.ro (M.S.S.); sorexmin@yahoo.com (M.P.); mihaela.danu@uaic.ro (M.D.); 2 “Olga Necrasov” Center of Anthropological Research, Romanian Academy—Iasi Branch, 700481 Iasi, Romania; 3 History and Etnography Museum of Târgu Neamț, 615200 Târgu Neamț, Romania; diavas_n82@yahoo.com

**Keywords:** archaeozoology, Middle Ages, NE Romania, paleoeconomy, Târgu Neamț sites

## Abstract

**Simple Summary:**

The authors present results on animal resources in the economy of medieval Moldova as they are reflected by analyses of animal skeletal remains recovered from archaeological sites situated in northeastern Romania and the Republic of Moldova. This paper aims to statistically evaluate the data of previous studies, and to analyse the case of the urban settlement from Târgu Neamț in the context of medieval Moldova. The results indicate the great importance of animal breeding and the low interest in hunting and fishing.

**Abstract:**

This study aims to contribute to the knowledge of the medieval Moldovan economy by evaluating animal resources (e.g., animal husbandry, hunting, fishing) based on the skeletal remains found in archaeologic sites from northeastern Romania and the Republic of Moldova. Animal remains, especially those from the urban settlement of the 14th–16th centuries from Târgu Neamţ (NE Romania), were described in terms of their frequencies (i.e., number of identified specimens and minimum number of individuals), morphometry, and livestock management (i.e., animal selection by age and sex). The results were compared with those obtained from other settlements—rural, urban, and fortress—from medieval Moldova. Correspondence analysis of the identified animals and settlements on the basis of the frequency values reveals associations between the two variables (animal species and settlement).

## 1. Introduction

The reconstruction of lifestyles and subsistence practices for past communities increasingly uses information provided by the study of biological remains discovered in archaeological sites, such as animal and plant remains [1,2,3,4,5,6,7,8]. Archaeozoological studies in medieval Europe have provided valuable information on various aspects of human–animal interactions, subsistence strategies, economic activities, and cultural practices [9,10,11,12,13,14], contributing to the understanding of medieval societies and their relationship with the animal world.

In the case of medieval Moldova, bioarchaeological research has become essential, since written documents are generally lacunary and ambiguous. There is much archaeological evidence for various activities in both rural and urban settlements, including those of securing animal and plant resources for food and other needs. Although archaeological research has provided information about the evolution of medieval urban centres, the focus has been on secular and ecclesiastical edifices and burial spaces, while aspects related to the anthropic impact on the environment and the evaluation of economic resources were superficially treated.

This study is focus on animal resources in the economy of medieval Moldova as they are reflected by archaeozoological analyses made for the territories of eastern Romania and the Republic of Moldova. This research asks questions about the roles of animals in the economy and diet of medieval Moldovan society, and whether the urbanisation was facilitated by the supply of good quality and quantity of meat (i.e., beef, mainly) from surrounding areas.

**Figure 1:**
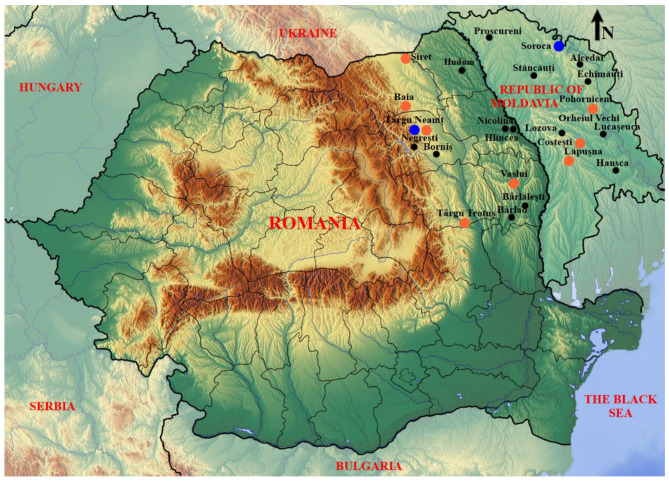
Settlements of medieval Moldova archaeozoologically analysed: urban in brown, rural in black, and fortress in blue.

Previous archaeozoological syntheses [15,16] generally discuss the faunal remains discovered in sites situated both to the right and left of the Pruth River which today separates the two studied countries (Figure 1). This paper aims to statistically evaluate the data presented by previous studies, and to analyse the case of the urban settlement of Târgu Neamț by referring to the general archaeozoological context of medieval Moldova.

The archaeozoological synthesis regarding the animal resources in the economy of medieval Moldova is based on samples of skeletal remains found in 28 sites, mainly provided by the literature.

## 2. Historical and Archaeological Context

The medieval state of Moldova appeared through the evolution of some pre-state nuclei that existed probably since the 13th century in the space between the Carpathian Mountains and the Dniester River, and which coagulated in a single formation as a natural process of unification, but also under the pressure of external factors, such as the danger of the expansion of the Hungarian Kingdom to the east, but also of the Golden Horde towards west. In the 14th century, to the east of Carpathian Mountains, a series of urban centres were established, also doubled by a defensive system made up of fortresses, which represented the foundation of the medieval state of Moldova (modern-day territories of eastern Romania and the Republic of Moldavia). These urban settlements have been studied mainly from the perspective of topography, architecture, and their evolution to becoming the cities of the modern period.

Documentary sources and archaeological research suggest that the genesis of urban settlements in medieval Moldova was a consequence of political transformations and economic development (e.g., craft activities, trade, and agricultural production), with both the local and foreign communities contributing to the evolution of towns. The inhabitants of the medieval Moldavian towns practiced plant cultivation and animal husbandry at the edges of settlements, also supplying themselves with animal and vegetable products from neighbouring villages or from other more distant areas.

Dimitrie Cantemir, in *Descriptio Moldaviae* (1716) [17], mentioned that, in Moldova, *Triticum* sp. (wheat) and *Hordeum vulgare* (barley) were mainly cultivated for both human and animal food. Apart from these cereals, the medieval population also grew *Panicum miliaceum* (millet), and to a lesser extent, *Avena sativa* (oats) and *Secale cereale* (rye). Medieval documents also recorded the cultivation of *Zea mays* (corn) since the middle of 18th century [18]. The cultivation of *Vitis vinifera* (grapevine) was especially intensified in the second half of the 16th century [17], with huge vineyards occupying the hills of Moldova. Extensive orchards of fruit trees in the medieval landscape of Moldova have been confirmed by the documents of the time.

The same author, Dimitrie Cantemir, points out that Moldavians were famous breeders of *Ovis aries* (sheep), *Bos taurus* (cattle), and *Equus caballus* (horse), domestic animals that were also exported to many parts of Europe. The cattle trade was especially intense in the 14th–17th centuries; large herds were recorded leaving Moldova [19].

**Figure 2:**
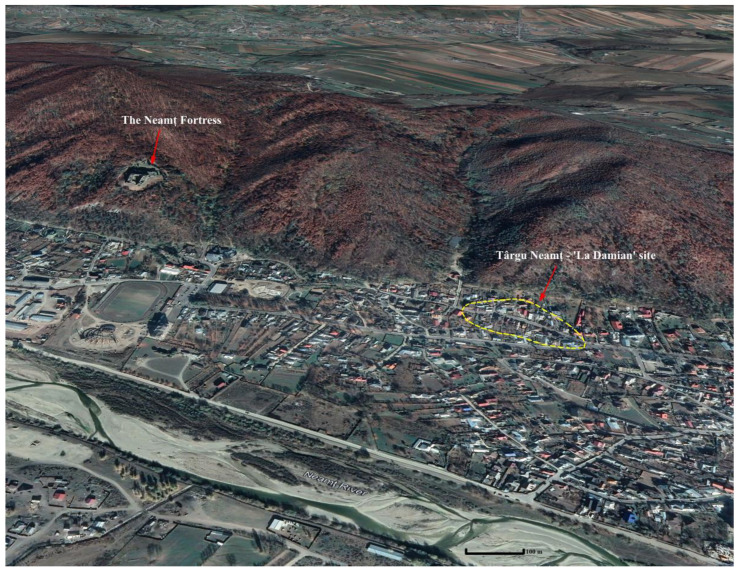
Location of the archaeological sites: Târgu Neamț–*La Damian* and Neamț Fortress.

The medieval urban centre of Târgu Neamț, located in the Subcarpathian area, which developed in the 14th century due to intense commercial activities, has been little known until recently, from the perspective of its archaeological vestiges [20,21]. Located at the intersection of important commercial routes, which connected Transylvania with Moldova, Târgu Neamț benefited from the existence in the immediate vicinity of an important fortification with a defensive role, known as the Neamț Fortress, built in the second part of the 14th century (Figure 2). Located on the terraces to the left of the Neamț River and bounded to the north by the Subcarpathian hills, the first urban core of Târgu Neamț was a small one, and due to the intense habitation until now, its archaeological investigation was very limited [22,23].

The medieval urban core of Târgu Neamț coincides with two archaeological sites which are very close to each other, but nevertheless present some chronological and structural differences: the Târgu Neamț–*La Damian* site (from which the faunal remains used in this work come) includes traces of three houses and household complexes of the 14th–15th centuries [23,24,25,26,27], and the Târgu Neamț–*La Canton* site includes traces of a church, a small cemetery, and a building wall dated to the 15th–16th centuries [26,28].

In the Târgu Neamț–*La Damian* site, the housing constructions were partially buried in the ground, as they are made of wood, with walls glued with clay; inside, they had fire installations, such as stoves made of tiles. In one of the houses (L3), investigated in 2021, the carbonized remains of leguminous plants (*Vicia faba*) and cereals (*Triticum aestivum, Hordeum vulgare,* and *Avena sativa)* were identified, providing valuable data about the habitat in the vicinity of the settlement and people’s eating habits [27]. The presence of cereals could be explained by their cultivation in the area adjacent to the settlement, most likely on the lower and middle terraces located to the right of Neamţ River. This hypothesis is supported by the existence of chaff and straw, identified in burnt plant material taken from the site. On the other hand, considering the fact that it is an urban site, the presence of cereals, also as a result of their procurement in commercial exchanges, cannot be excluded. The context in which the grains were identified (all in the same container, so in a mixture) suggests that they were used to obtain flour. The significant presence of the bean means that this legume was also used for food. The anthracological remains discovered inside the house indicate the presence of a forest area nearby, which is plausible if we consider the proximity of the Pleșu Peak which, most likely, was also wooded during the Middle Ages. In addition to beech, remains of *Robinia pseudoacacia* (acacia) were identified, as well as remains of conifers, possibly *Abies alba* (fir).

Based on the archaeobotanical remains recovered from the L3 dwelling, interesting observations related to the climate of that period were also made; a cooling episode was recorded. It was also possible to appreciate that, in the vicinity of the settlement, there was a landscape that combined the forest areas dominating the highlands from the north with grassy and even marshy areas, which corresponded to the former meadow area of the Neamt River [29].

The Neamț Fortress was built in the 14th century and functioned until the first decades of the 18th century [30]. Inside the fortress, archaeological research was carried out in several periods [31,32,33,34], but the results were only partially valorised, the focus being on the constructive stages and defensive arrangements. During recent archaeological excavations [35], several chronological levels were investigated, and animal skeletal materials were recovered from those attributed to the 14th–16th centuries. As it is a construction with a military role, the archaeozoological study indicated, on the one hand, the consumption behaviours of those who were stationed inside the fortress, and on the other hand, data can be obtained regarding the supply method and the relationship between the fortification and the nearby urban settlement.

## 3. Material and Methods

This work uses sets of archaeozoological data taken from published studies (Table 1), as well as unpublished data obtained from the analysis of two samples of faunal remains from the sites of Târgu Neamț–*La Damian* and Neamț Fortress. The sites from which the archaeozoological data analysed in this paper come from represent rural and urban settlements, also fortresses, distributed in different geographical units of medieval Moldova, as can be seen in Figure 1 (i.e., Moldavian Plateau, Moldavian Plain, and Subcarpathians). Table 1 shows the types of settlements, most of them being rural (in number of 18), nine urban sites, and two fortresses (Soroca Fortress having analysed two levels).

The animal skeletal remains discovered in the Târgu Neamț–*La Damian* site come from recent research carried out in 2015–2021, and those of the Neamț Fortress resulted from archaeological excavations carried out in 2007–2008, during the rehabilitation works of the monument.

Archaeozoological analysis was conducted in the Laboratory of Archaeozoology, Faculty of Biology, Alexandru Ioan Cuza University of Iaşi, Romania, and it consisted of anatomical and taxonomic identifications, taphonomic evaluation, quantification, estimations of age at slaughter, sex, and osteometry.

For the anatomical and taxonomic identifications, the comparative osteological collections from the Laboratory of Archaeozoology were used, as well as specialized works for the differentiation of some close taxa, such as sheep (*Ovis aries*) and goat (*Capra hircus*) [36]. In the taphonomic evaluation, different types of traces preserved on the bone fragments were evaluated and identified, such as those of anthropic origin (trapping, burning, and processing) and/or animal (gnawing traces by dogs) [37,38]. The quantification of the samples was based on the number of identified specimens (NISP) and the minimum number of individuals (MNI) estimated for the identified mammal species [39].

The estimation of slaughter ages was carried out both on the basis of the dentition and the postcephalic skeleton, using data provided in the works by Udrescu et al. [38], Barone [40], and Reitz and Wing [39]. The mature/immature separation made in the case of domestic animals also took into account the age of sexual maturity; this limit is 2.5 years for cattle, 1.5 years for sheep/goat, and 13 months for pig [29]. Sex estimation was performed only for cattle, based on the morphometric characteristics of the metapodials. The variation ranges of the metapodial indices for the cattle sexes (i.e., female, male, or castrated) were taken from Udrescu et al. [38].

The measurements respected the A. von den Driesch guide [41], and the resulting data were used to separate domestic and wild forms (i.e., *Sus domesticus*/*Sus scrofa*), to estimate the sex in *Bos taurus*, and the withers height in different mammal species (i.e., *Bos taurus*, *Ovis aries*, and *Sus domesticus*). Different coefficients were used to calculate the withers height, as follows: the coefficients of Fock [42] for *Bos taurus*, those of Teichert [43] for *Ovis aries*, and the coefficients of Teichert [44,45] for *Sus domesticus*.

Statistical multivariate analyses, such as the correspondence analysis (CA), are widely used in bioarchaeology studies, including archaeozoology. These analytical methods are especially useful for considering multiple variables (e.g., archaeological sites, settlements, and time periods), leading to the interpretation of bioarchaeology data in a much broader context [46].

Correspondence analysis is an extension of principal component analysis, suitable for exploring relationships between qualitative variables and categorical data (in contingency tables, rows, and columns) [47]. The method allows, in the case of our study, the codependent analysis of the data related to the context/archaeological site or historical periods, with those related to the abundance and frequency of faunal remains identified in the archaeological sites [48]. The advantage of this method is that the mathematical results can be summarized in two-dimensional graphic representations, which are easy to follow and interpret. The interpretation of the correspondence between the variables is related to the significance test of the correlation/association between them. For this purpose, we used the chi-squared test, with a significance threshold of *p* < 0.05 and total inertia. The chi-squared test is a statistical hypothesis test used in the correspondence analysis. This test is used for independent testing between the rows and columns of the continence table (in our study, between settlements and identified animal species). Total inertia can be computed as the weighted sum of the squared distances of the rows or the columns to their respective barycentre. In correspondence analysis, the total variance (often called inertia) of the factor scores is proportional to the independent chi-square statistic.

The correspondence analysis was performed for the following datasets: (a) frequency of animal species in urban settlements, (b) frequency of animal species in rural settlements, and (c) frequency of animal species in fortress settlements.

Significance testing was applied to verify the significance of the frequency differences in the three types of settlements (i.e., urban, rural, and fortress) for each individual species. The one-way analysis of variance (ANOVA) was used to find whether there were any statistically significant differences between the mean frequencies of the three settlement types [49,50].

**Table 1:** Archaeozoologically analysed settlements.

Chronology	Type of Settlement	Settlement [Reference]	Sample Size (NR = Number of Remains)
14th–17th	urban	Baia [51,52,53,54,55,56,57]	8415
14th–17th	urban	Siret [52,58,59]	5833
15th–16th	urban	Târgu Neamț (This paper)	1375
15th–16th	urban	Vaslui [60]	1900
14th–17th	urban	Târgu Trotuş [52]	510
14th	urban	Old Orhei (Golden Horde) [61]	414
15th–17th	urban	Old Orhei (15th–17th centuries) [61]	1365
14th	urban	Costești (This paper)	4892
14th–17th	urban	Lăpușna (This paper)	2143
14th–16th	fortress	Neamț Fortress (This paper)	465
15th–16th	fortress	Soroca Fortress [62]	1017
17th–18th	fortress	Soroca Fortress [62]	625
14th	rural	Hudum [63]	128
12th–13th	rural	Nicolina [64]	47
14th–15th	rural	Hlincea [65]	152
13th–14th	rural	Bârlad [66]	800
10th–11th	rural	Bârlăleşti [67]	1300
14th–17th	rural	Borniş-Obarsia [68]	850
14th–15th	rural	Borniş-Malesti [68]	450
14th–18th	rural	Borniş-Siliste [68]	774
17th–18th	rural	Negreşti [65]	323
6th–12th	rural	Proscureni [69]	1691
8th–14th	rural	Pohorniceni [69]	1175
10th–14th	rural	Hansca [69]	5999
9th–15th	rural	Lucaseuca [69]	796
14th	rural	Lozovo [69]	373
15th–18th	rural	Stancauti [69]	67
9th–10th	rural	Alcedar [69]	990
9th–10th	rural	Echimauti [69]	3911
9th–11th	rural	Calfa [69]	100

## 4. Results and Discussion

**Case study of the urban and fortress settlements from Târgu Neamț (NE Romania)**

The archaeozoological sample from the urban site of Târgu Neamt–*La Damian*, as well as the one from Neamț Fortress, contain household remains, as indicated by the numerous identified butchering and burning traces on the bone fragments (Table 2). A small number of bone fragments have traces of gnawing by other animals, especially dogs, and two pieces from the urban sample were processed (i.e., two fragments of red deer antlers). Both analysed samples contain a variety of taxa—fish, birds, and mammals—the latter ones being dominant and representing about 97%, and 98%, respectively (Table 3).

As a result of the anatomical and taxonomic identifications, three main categories of animal resources were highlighted, which, expressed in terms of the local economy, are: animal husbandry, hunting, and fishing.

**Figure 3:**
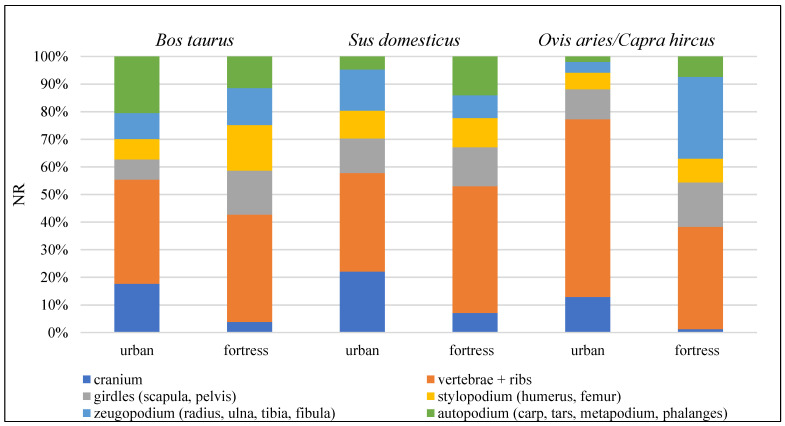
Anatomical distributions of skeletal remains for cattle (*Bos taurus*), pig (*Sus domesticus*), and sheep/goat (*Ovis aries*/*Capra hircus*).

Animal husbandry represented a basic source of food, both in the town and in the fortress; domestic mammals were mainly consumed, but, to a lesser extent, domestic birds were as well. Of the total bird remains identified in the urban sample, 23 belonged to hens (*Gallus domesticus*); only 2 hen bones were found in the sample from the fortress. Instead, the skeletal remains from domestic mammals are much more numerous and taxonomically varied; their quantification can be found in Table 4. The domestic mammals with the highest frequency in both samples were cattle (*Bos taurus*), pig (*Sus domesticus*), and sheep/goat (*Ovis aries*/*Capra hircus*), but the ratio between them slightly differs. Thus, cattle appear dominant as NISP in both samples, but as MNI only in the urban site; pig follows in frequency in both samples, both as NISP and as MNI in the urban site; and sheep/goat are on third place as NISP, and just over pig as MNI in the fortress sample (Table 4). The anatomical distribution of the skeletal remains, from these domestic species with higher frequencies (i.e., cattle, pig, and sheep/goat), shows the presence of all body regions in both samples, which would indicate the use of the whole animal carcasses both within the urban settlement and in the fortress (Figure 3).

The assessment of the selection by age for these three main domestic taxa is based on the characteristics of both the dentition and bones from the postcephalic skeleton. As can be seen in Table 5, in cattle, there is a predominance of individuals slaughtered at a mature age, in both samples. Individuals slaughtered at maturity also predominate, but not so much, in sheep/goat, as well as pig in the urban site (Table 5). Regarding the sex estimation, it was realized only for cattle, based the whole preserved metapodials, which were much more numerous in the urban sample (Table 6). Thus, in the urban site, 20 female, 1 male, and 6 castrated animals were evaluated based on metapodials, while in the fortress, only 1 female and 1 male were identified.

From the multitude of osteometric data taken, those of preserved whole bones draw our attention, as they can confirm the withers heights estimations for the main domestic species (Table 6). In cattle, the withers height was differentiated according to sex: in the urban sample, averages of 111 cm and 116 cm were obtained for females and castrates, respectively, and only one value of 125 cm was identified for males; in the fortress, only values of 120 cm and 114 cm, respectively, were identified for a female and a male. In pig, similar values of the withers height were obtained for both the samples, with an average of about 78 cm. In the case of the sheep, there were estimates of body size only from the fortress, with an estimated average of about 73 cm.

The horse (*Equus caballus*) skeletal remains, although they had a low frequency in both samples (Table 4), showed clear butchery traces, indicating the consumption of this species’ meat; these traces were identified on four bone fragments from the urban sample (i.e., pelvis, radius, tibia, and mandibula), and on six from the fortress (i.e., two ribs, three radii, tibia, and metapodium). Dog (*Canis familiaris*) remains also had low frequencies in both the samples (Table 4); no traces of cutting were identified at all. Both the horse and dog skeletal remains indicate mature ages (Table 5).

Hunting is seldom represented in both samples; it was evaluated mainly based on wild mammal remains (all indicating mature ages, Table 5), to which we also added the very few bones of birds not identified by species but assumed to have been wild. The game species were similar in both sites (Table 4), except for hare (*Lepus europaeus*), which did not appear in the fortress; this absence could be attributed to the small size of the sample. Table 4 shows that red deer (*Cervus elaphus*) was the favourite game, followed by wild boar (*Sus scrofa*), and then by hare in the urban site, or by roe deer (*Capreolus capreolus*) in the fortress.

As shown in Table 3, fish remains were very rare in both samples; their frequency was probably underestimated due to the non-sieving of the archaeological sediments. The fish remains belonged to the teleostean group, but they were not identified by species except for a single bone from the fortress attributed to catfish (*Silurus glanis*).

Following the detailed comparative analysis made of the two sites in Târgu Neamţ, urban and fortress, it was found that there were no important differences in terms of general resources of animals for consumption (i.e., domestic and hunted animals, and fish), in the livestock management or morphometric characteristics. Except for hare (*Lepus europaeus*), which was not identified in the fortress, the list of domestic and wild mammals was identical for the two samples. However, there was a difference regarding the ratio between the main domestic species, in the favour of cattle as both NISP and MNI in the urban site, while in the fortress, sheep/goat and pig appeared predominant as MNI. If we compare the fortress of Neamţ to that of Soroca, we find similarities in the structure of the samples by species, but only for the 14th/15th–16th centuries; later, in the 17th century, there was a clear change in the Soroca fortress, in which pig had the highest frequency as NISP, probably corresponding to the Polish occupation [70].

**Figure 4:**
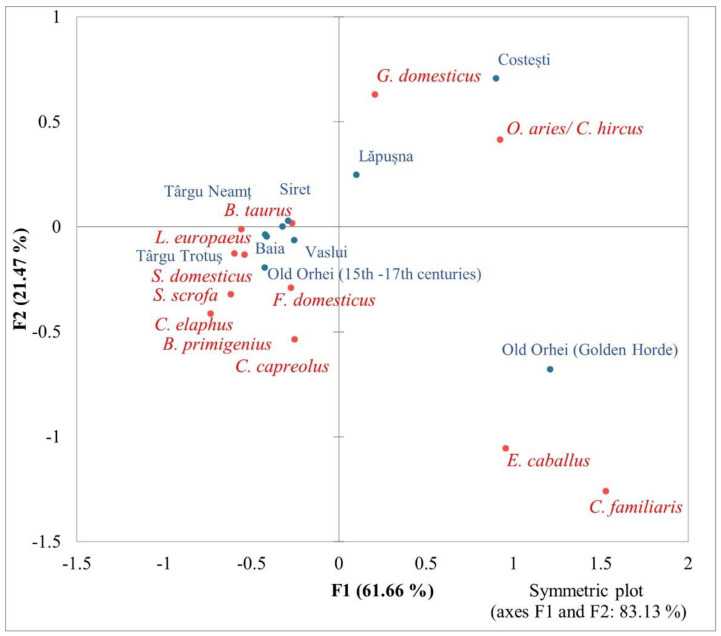
Correspondence analysis of identified animal species and urban settlements.

As Figure 4 shows, the site of Târgu Neamţ–*La Damian* is similar to other urban sites of medieval Moldova (i.e., Baia, Siret, Târgu Trotuş, Vaslui, and Old Orhei in the 15th–16th centuries) in terms of the frequency of some identified species. Thus, the preference for the consumption of beef in these medieval Moldovan towns is notable. But the cattle-breeding strategy was focused not only on obtaining meat, but also on providing products considered secondary (obtained without slaughtering the animal), such as dairy, traction force, and reproductive stock. In fact, obtaining such secondary products was a priority for the urban community, since the selection of animals followed the preservation of a stock of adult specimens consisting of numerous females, a few castrated animals, and a male. Similar results regarding the selection of cattle according to age and sex were also mentioned for other medieval cities in Moldova [71]. The averages of withers height in cattle in the Târgu Neamţ–*La Damian* site fell within the range in variation estimated for the whole medieval Moldova, of 97–135 cm [15]. The average of the cattle withers height in medieval Moldova was found to be among the highest in Europe [15], according to the data provided by Audoin-Rouzeau [72].

Pig appeared in second place as a preference for consumption in the Târgu Neamţ–*La Damian* site, similar to other Moldavian urban settlements. The number of individuals butchered after the age of 13 months was over half of the total estimated, like in other urban settlements [71], indicating a slower growth rate so that slaughtering was profitable at maturity. The average height at the withers, about 78 cm, was relatively high; placing it in the range in variation estimated for medieval Moldova, of 70–83 cm [15]. Sheep/goat followed pig in frequency at the Târgu Neamţ–*La Damian* site, and in other urban settlements in medieval Moldova (i.e., Baia, Siret, Târgu Trotuş, Vaslui, and Old Orhei in the 15th–16th centuries), and the estimated ages at slaughter indicate a mixed exploitation: for both meat and secondary products (e.g., milk, wool). Osteometric data obtained for the sheep indicate relatively large withers heights at Târgu Neamţ (Table 6), in the variation range estimated for medieval Moldova, of 61–77 cm [15].

The horse remains analyses from the Târgu Neamț, all belonging to adult individuals, show that this domestic species was occasionally consumed, despite religious interdictions and symbolic/emotionally connection between humans and horses. Although no osteometric data were obtained from the samples of Târgu Neamţ necessary to estimate the horse body conformations (i.e., withers hight and gracility), from previous studies, it is known that individuals of sub-medium and medium sizes (128–136 cm and 136–144 cm, respectively) were more common in medieval Moldova, but larger sizes over 150 cm have also been identified [15].

In the short list of wild animals identified in the samples of Târgu Neamţ, red deer and wild boar had a relatively higher frequency among the game mammals, as in many other settlements of medieval Moldova [15]. Hunting in the Middle Ages must be viewed from a perspective of social rules; restricted to the nobility.

**Animal resources in the economy of medieval Moldova**

As indicated by previous studies [15], animal husbandry had a major importance in the analysed settlements; the local economies were focused on the breeding of cattle (*Bos taurus*), pig (*Sus domesticus*), and sheep/goat (*Ovis aries*/*Capra hircus*).

Other animal resources identified archaeozoologically, but with a lower frequency in terms of the number of remains, are represented by hunting and fishing. In most of the analysed sites, wild mammals are recorded in small proportions, which is in fact a general characteristic for many regions of Europe in the Middle Ages [9,13,15,73]. As hunted animals, species of large sizes are better represented, such as the following: red deer (*Cervus elaphus*), wild boar (*Sus scrofa*), roe deer (*Capreolus capreolus*), and even aurochs/bison (*Bos primigenius*/*Bison bonasus*). Fishing appears even less represented [15], not only due to the reduced importance in the food economy of the analysed settlements, but also due to the fact that the sieving of the archaeological sediments was not carried out in order to recover all the small faunal remains. This is a limitation of the archaeozoological studies for medieval Moldova, which affects, by underestimation, not only the fish remains but also those of birds and mammals of small sizes.

In a previous synthesis study [15], it was shown that the frequency of the identified animal species varied according to geographical, ethnic, and religious factors. It was found that cattle were constantly dominant in almost all the analysed samples, and regions specialized in their growth were even identified, such as the north and centre of medieval Moldova. In the peri-Carpathian region, pig breeding was more important, while in arid flat lands, sheep/goat appeared more frequently. In the sites of Mongolian origin (i.e., Old Orhei and Costești), the absence of pig remains was highlighted, as was an increased frequency of sheep/goat and cattle.

In the present study, the statistical analysis considered the type of settlement (i.e., rural, urban, and fortress), which could influence the distribution of animal species mainly as food resources.

The correspondence analysis of the identified animals and urban settlements on the basis of the frequency values reveals significant associations between the two variables (i.e., animal species and settlement) (chi-squared test: 476; *p*-value: 0.0001; total inertia 0.54) (Figure 4). Results show that the first two dimensions (axes) added up explain over 83% of the data scatter, which is quite notable and enables reasonable interpretations (F: 61.66; F2: 21.47%). We noticed a high degree of similarity/correspondence between the urban settlements of Baia, Siret, Vaslui, Târgu Trotuş, Târgu Neamț, and Old Orhei (15th–17th centuries) (chi-squared distances <0.6), and this result is attributed mainly to the high frequencies, and to the significant correlations between the animal species identified in these samples. Most of the identified species are displayed within or nearby the lower left quadrant (with negative values on the F1 axis), both domestic (i.e., *Bos taurus*, *Sus domesticus*, and *Felis domesticus*) and wild (i.e., *Lepus europaeus*, *Sus scrofa*, *Cervus elaphus*, *Bos primigenius*, and *Capreolus capreolus*). The scattering on the positive axis (F1) (upper right quadrant) of the Lapusna and Costesti (Mongolian city) settlements is due to the great correlation with sheep/goat (*Ovis aries*/*Capra hircus*) and hen (*Gallus domesticus*). A particular position appears in the case of the Mongolian city Old Orhei (Golden Horde), for which, in addition to the association with sheep/goat, there is an even stronger closeness to horse (*Equus caballus*) and dog (*Canis familiaris*).

**Figure 5:**
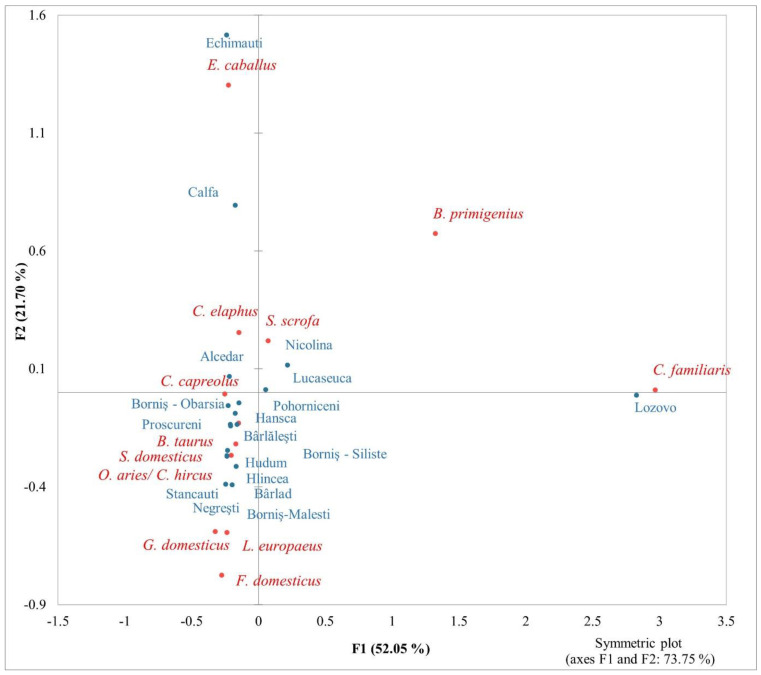
Correspondence analysis of identified animal species and rural settlements.

In the Figure 5, illustrating the correspondence between the identified animal species and the rural settlements, there are some statistically significant relationships (chi-squared test: 165, *p*-value < 0.0001; total inertia: 0.93). As in the previous analysis, the distribution along the F1 axis mostly explains the correspondence between the involved variables (52.05%). Axis 2 explains the variability in archaeozoological samples in the proportion of 21.7%. The dispersion of the identified species and the rural sites, especially on the F1 axis (with negative scores), is notable. The sites of Echimăuți and Lozovo, both in the current Republic of Moldavia, appear more distant from most of the others, and more strongly correlated with the presence of horse and dog, respectively.

**Figure 6:**
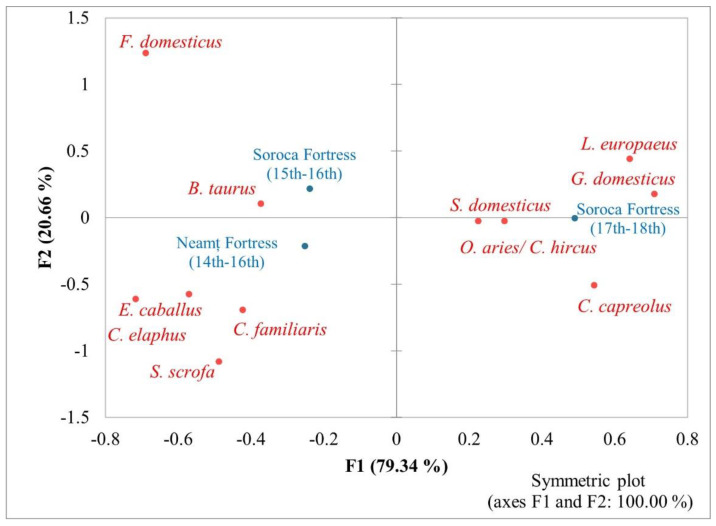
Correspondence analysis of identified animal species and fortresses.

The correspondence analysis of the animal species identified in the three fortresses is presented in Figure 6. Axis 1, which represents 79.34% of the entire variability, and F2 with 20.66%, highlight quite well the relationships between species and fortresses. We noticed the statistically significant relationships (chi-squared test: 45.32 *p*-value < 0.0001; total inertia 0.93) between the identified wild and domestic animals and the three fortresses (chi-squared test = 34.7; *p* = 0.007). The Figure 6 shows a particular association between cattle (*Bos taurus*) and the Soroca Fortress in the period of the 15th–16th centuries (arranged on negative F1, upper quadrant), and also different affinities appear later between the same fortress in the 17th–18th centuries and pig (*Sus domesticus*), sheep/goat (*Ovis aries*/*Capra hircus*), hen (*Gallus domesticus*), and hare (*Lepus europaeus*); the last ones are dispersed in the upper right quadrant (positive scores on the F1 axis).

**Figure 7:**
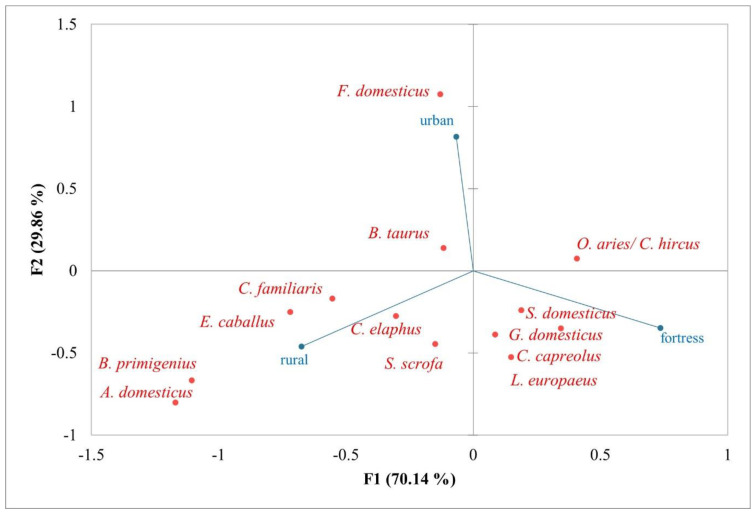
Correspondence analysis of identified animal species and settlement type.

**Figure 8:**
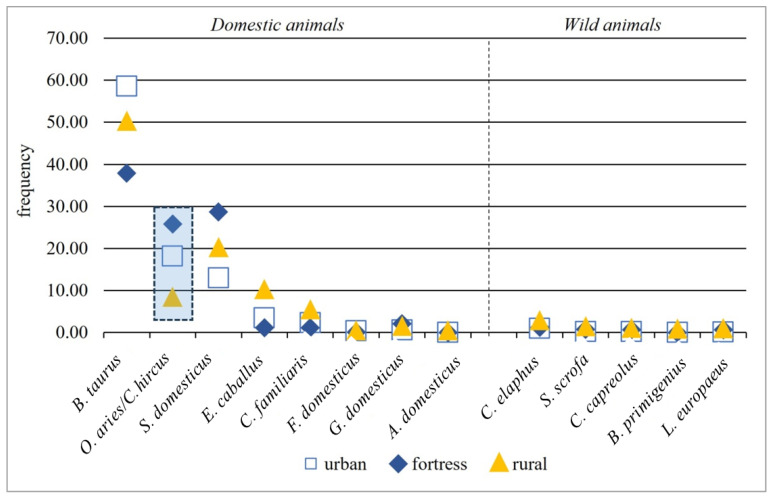
Domestic and wild animals in rapport with the settlement types (blue area: statistically significant differences between frequencies).

The correspondence for the identified animal species and the type of settlement (i.e., urban, rural, and fortress) is represented in the Figure 7 and Figure 8. In Figure 7, the relationships between identified animal species and the type of settlements are statistically insignificant (chi-squared test: 34.4; *p*-value: 0.072; total inertia: 0.117). However, it is worth mentioning an affinity of cattle (*Bos taurus*) and cat (*Felis domesticus*) for the urban settlements, and also that of medium-sized species, both domestic (i.e., *Ovis aries*/*Capra hircus*, *Sus domesticus*, and *Gallus domesticus*) and wild (i.e., *Capreolus capreolus* and *Lepus europaeus*), for the fortresses (Figure 7). It is noted on the graph, in the lower left quadrant, the correspondence between large-sized hunted species (i.e., *Cervus elaphus*, *Sus scrofa*, and *Bos primigenius*) and rural settlements.

Although in Figure 8 and Table 7 a differentiation according to consumption preferences (i.e., cattle are more important in urban areas, pig in fortresses and rural sites, sheep/goat in fortresses and urban areas, and horse in rural sites) can be distinguished, it should be mentioned that statistically significant differences were only found in the sheep/goat (*Ovis aries*/*Capra hircus*) frequency between the rural and fortress settlements (one-way ANOVA, F: 4.58; *p*: 0.0004). Statistically, *Bos taurus* is one of the animal species that does not differ significantly in frequency between the three types of settlements (one-way ANOVA, F: 1.71; *p*: 0.096).

## 5. Conclusions

This paper presents a synthesis of archaeozoological data obtained for different types of settlements from medieval Moldova. Analysing separately the archaeozoological sample from the Târgu Neamţ–*La Damian* urban site, we found obvious similarities with the group of urban settlements mentioned previously, constituting a compact group in the diagram in Figure 4. The exploited animal resources were limited to fish, birds, and mammals. Most of the analysed skeletal remains belonged to mammals, especially domestic animals (i.e., cattle, sheep, goat, pig, horse, and dog). Hunting had a small importance, with a low diversity of identified wild species (i.e., red deer, wild boar, roe deer, and hare), reflecting mainly the exploitation of forested environments.

The frequency of animal species identified in 30 samples from medieval urban, rural, and fortress sites was statistically analysed. Correspondence analysis was firstly used separately for each settlement group (i.e., urban, rural, and fortress). Thus, it was found that the group of urban settlements was quite compact in terms of use of animal resources for consumption (especially cattle and pig), with the exception of the Mongolian cities of Costesti and Old Orhei, which were strongly associated with sheep/goat, and also with horse and dog in the case of Old Orhei. The rural settlements, more numerous, were also compactly distributed, associated especially with cattle, pig, and sheep/goat. Three separate sites appeared to be associated more strongly with horse (Echimauti and Calfa) or with dog (Lozovo), without evidence of consumption for the latter. A bias could also be detected in the distribution of the fortresses. There is a group of two similar ones (Neamţ Fortress and Soroca Fortress—15th–16th centuries), associated with an increased frequency of cattle, and separately, the later Soroca Fortress (17th–18th centuries) was associated with a higher consumption of pork, attributed to the Polish occupation. When the statistical analysis was conducted to differentiate the types of settlements according to the preference for certain animal resources, no significant differences appeared, which means that the urban environment was still very similar to the rural one in terms of food resources of animal origin. However, future statistical studies on larger and more numerous samples of faunal remains will perhaps confirm that the urbanization assumed an intense supply of meat, especially from cattle, provided from the neighbouring areas.

Looking in general at the animal resources used in the economy of medieval Moldova, similarities with neighbouring regions can be noted, such as the importance of animal husbandry (mainly cattle, pig, and sheep/goat), sustained by the plant cultivation (especially cereals) and the low rate of hunting. The influence of the Mongol Empire is noticeable in the eastern area of medieval Moldavia, the area where large Mongolian urban settlements were developed in the 14th century, with a predominant consumption of sheep/goat meat.

## Figures and Tables

**Table 2:** Distribution of remains with taphonomy evidence (NISP = number of identified specimens).

Taphonomy Evidence	Târgu Neamț–*La Damian*		Neamț Fortress	
	NISP	%	NISP	%
Remains with butchering traces	563	40.95	260	55.91
Remains with burn traces	34	2.47	8	1.72
Remains with animal teeth marks	57	4.15	17	3.65
Manufactured antlers	2	0.15	0	0
Total sample	1375		465	

**Table 3:** Distribution of animal remains by taxonomic group (NISP = number of identified specimens).

Taxonomic Group	Târgu Neamț–*La Damian*		Neamț Fortress	
	NISP	%	NISP	%
Fish	7	0.51	2	0.43
Birds	33	2.40	6	1.29
Mammals	1335	97.09	457	98.28
Total sample	1375	100.00	465	100.00

**Table 4:** Quantification of mammal remains (NISP = number of identified specimens, MNI = minimum number of individuals).

Taxon	Târgu Neamț–*La Damian*				Neamț Fortress			
	NISP	%	MNI	%	NISP	%	MNI	%
*Bos taurus* (cattle)	798	70.68	22	44.00	157	43.49	4	15.38
*Sus domesticus* (pig)	168	14.88	13	26.00	85	23.55	6	23.07
*Ovis aries*/*Capra hircus* (sheep/goat)	101	8.95	5	10.00	81	22.44	7	26.92
*Equus caballus* (horse)	16	1.42	2	4.00	9	2.49	2	7.69
*Canis familiaris* (dog)	6	0.53	1	2.00	9	2.49	2	7.69
Total domestic mammals	1089	96.46	43	86.00	341	94.46	21	80.77
*Cervus elaphus* (red deer)	21	1.86	3	6.00	10	2.77	2	7.69
*Sus scrofa* (wild boar)	10	0.89	1	2.00	7	1.94	2	7.69
*Lepus europaeus* (hare)	8	0.71	2	4.00	0	0	0	0
*Capreolus capreolus* (roe deer)	1	0.09	1	2.00	3	0.83	1	3.84
Total wild mammals	40	3.54	7	14.00	20	5.54	5	19.23
Total identified mammals	1129	100	50	100	361	100	26	100
Unidentified mammals	206	-	-	-	96	-	-	-
Total mammals	1335	-	-	-	457	-	-	-

**Table 5:** Estimated age of slaughter for the identified mammal species (MNI—minimum number of individuals).

Species	Târgu Neamț–*La Damian*			Neamț Fortress		
	Total MNI	Immature	Mature	Total MNI	Immature	Mature
*Bos taurus*	22	3	19	4	1	3
*Sus domesticus*	13	5	8	6	3	3
*Ovis aries/Capra hircus*	5	2	3	7	3	4
*Equus caballus*	2	0	2	2	0	2
*Canis familiaris*	1	0	1	2	0	2
*Cervus elaphus*	3	0	3	2	0	2
*Sus scrofa*	1	0	1	2	0	2
*Lepus europaeus*	2	0	2	0	0	0
*Capreolus capreolus*	1	0	1	1	0	1

**Table 6:** Withers height and sex estimations based on osteometric data.

Species	Anatomical Element	Bone Length (mm)	Withers Height (mm)	Sex Estimation
**Târgu Neamț–*La Damian***				
*Bos taurus*	metacarpus	194	1164	female
*Bos taurus*	metacarpus	192	1155	female
*Bos taurus*	metacarpus	186	1116	female
*Bos taurus*	metacarpus	192	1155	female
*Bos taurus*	metacarpus	186	1116	female
*Bos taurus*	metacarpus	195	1170	female
*Bos taurus*	metacarpus	179	1074	female
*Bos taurus*	metacarpus	181	1086	female
*Bos taurus*	metacarpus	195	1173	female
*Bos taurus*	metacarpus	162	975	female
*Bos taurus*	metacarpus	193	1158	female
*Bos taurus*	metacarpus	184	1104	female
*Bos taurus*	metacarpus	195	1170	female
*Bos taurus*	metacarpus	180	1080	female
*Bos taurus*	metacarpus	197	1208	castrated
*Bos taurus*	metatarsus	203	1106	castrated
*Bos taurus*	metatarsus	216	1177	castrated
*Bos taurus*	metatarsus	210	1147	castrated
*Bos taurus*	metatarsus	216	1177	castrated
*Bos taurus*	metatarsus	214	1166	castrated
*Bos taurus*	metatarsus	204	1091	female
*Bos taurus*	metatarsus	206	1102	female
*Bos taurus*	metatarsus	199	1064	female
*Bos taurus*	metatarsus	210	1123	female
*Bos taurus*	metatarsus	204	1094	female
*Bos taurus*	metatarsus	216	1155	female
*Bos taurus*	metatarsus	225	1251	male
*Sus domesticus*	metacarpus 4	81	823	-
*Sus domesticus*	metacarpus 4	77	781	-
*Sus domesticus*	metatarsus 3	80	752	-
**Neamț Fortress**				
*Bos taurus*	metacarpus	201	1206	female
*Bos taurus*	metatarsus	206	1143	male
*Sus domesticus*	metacarpus 3	68	700	-
*Sus domesticus*	metacarpus 4	83	849	-
*Sus domesticus*	metacarpus 4	77	781	-
*Sus domesticus*	metatarsus 4	97	853	-
*Sus domesticus*	calcaneus	76	735	-
*Ovis aries*	astragalus	33	748	-
*Ovis aries*	astragalus	31	714	-

**Table 7:** Domestic and wild animals in rapport with the settlement types (% mean of frequencies and standard errors).

Species	Urban		Fortress		Rural	
	Mean	Std. Error	Mean	Std. Error	Mean	Std. Error
*Bos taurus*	58.61	6.28	37.89	10.3	49.81	3.9
*Ovis aries/Capra hircus*	18.15	6.4	25.76	4.1	8.00	1.1
*Sus domesticus*	12.99	2.8	28.68	6.03	19.84	2.9
*Equus caballus*	3.48	1.7	1.17	0.67	9.80	3.1
*Canis familiaris*	2.32	1.5	1.15	0.66	5.05	3.6
*Felis domesticus*	0.44	0.35	0.03	0.03	0.05	0.04
*Galus domesticus*	0.57	0.26	2.05	1.05	1.08	0.4
*Anser domesticus*	0.00	0	0.00	0	0.01	0
*Cervus elaphus*	0.99	0.6	1.22	0.8	2.42	0.7
*Sus scrofa*	0.26	0.1	0.72	0.6	1.01	0.3
*Capreolus capreolus*	0.22	0.1	0.68	0.35	0.60	0.1
*Bos primigenius*	0.03	0.01	0.00	0	0.42	0.2
*Lepus europaeus*	0.10	0.07	0.63	0.34	0.52	0.28

## Data Availability

Data sharing is not applicable to this article.
